# Healthcare Providers’ Perspectives on Generative Artificial Intelligence (GenAI) Adoption, Adaptation, Assimilation, and Use in the United States

**DOI:** 10.3390/healthcare14060775

**Published:** 2026-03-19

**Authors:** Obinna O. Oleribe, Marissa Brash, Adati Tarfa, Ricardo Izurieta, Simon D. Taylor-Robinson

**Affiliations:** 1 Department of Health Sciences, School of Public Health and Health Sciences, California State University, Carson, CA 90747, USA; 2 Center for Family Health Initiative (CFHI), Orange, CA 92865, USA; 3 Department of Public Health, College of Nursing and Health Sciences, Azusa Pacific University, Azusa, CA 91702, USA; 4 Department of Internal Medicine, Yale School of Medicine, New Haven, CT 06520, USA; 5 Department of Surgery and Cancer, St Mary’s Hospital Campus, Imperial College London, London W2 1NY, UK; s.taylor-robinson@imperial.ac.uk

**Keywords:** generative artificial intelligence, healthcare, provider perspectives, adoption and integration, challenges and opportunities

## Abstract

**Highlights:**

**What are the main findings?**

- Most U.S. clinicians in the study perceive GenAI as useful in patient care, with confidence increasing over time, indicating strong momentum for adoption.
- Formal GenAI training and organizational adoption remain limited, creating a misalignment between clinician interest and system-level preparedness.
- Clinicians primarily value GenAI for documentation efficiency and error reduction, while barriers center on limited AI literacy and workforce displacement fears.

**What are the implications of the main findings?**

- Healthcare organizations must prioritize standardized, role-specific training to convert positive sentiment into safe and effective use.
- Successful integration will require transparent governance, clinician oversight, and clear accountability to address ethical and trust concerns.
- Healthcare administrators should intentionally reinvest productivity gains into strengthening patient–provider relationships, improving work–life balance, and reducing clinician burnout.

**Abstract:**

**Background:** Generative artificial intelligence (GenAI) is rapidly permeating healthcare; yet, U.S. clinicians still report mixed feelings about its reliability, impact on workflow, and ethical implications. Current data on provider sentiment are needed to guide safe, patient-centered AI implementation in healthcare. Objective: This study aimed to assess U.S. healthcare providers’ perceptions of generative AI adoption, perceived usefulness, training needs, barriers, and strategies for safe integration. **Methods:** A nationwide, IRB-approved, cross-sectional survey was administered to healthcare professionals using Qualtrics. A convenience sample of clinicians was recruited via professional listservs and e-mail invitations. The 20-page questionnaire captured demographics, GenAI exposure, organizational adoption status, perceived usefulness (5-point scale), barriers, and mitigation strategies. SPSS v27 and Microsoft Excel were used for statistical analysis. **Results:** Of 130 respondents, 109 completed the core survey (completion rate 83.8%). Participants were 38.5% physicians, 16.5% nurses, 12.8% allied professionals, and 32.2% other providers; 54.2% were women, and 64.8% were ≥50 years. Overall, 86.9% agreed that GenAI is useful in current patient care, rising to 92.9% when asked about future usefulness. Only 42.4% had received formal GenAI training, and just 23.2% reported that their organization had begun adopting AI. The top perceived benefits were improved documentation/clerking (57.0%) and error reduction (49.4%). Dominant barriers included limited AI knowledge (24.7%) and fear of job loss (16.9%). Despite concerns, 72% expressed willingness to support broader GenAI adoption, favoring human oversight (67.1%) and staff training (60.8%) as key safeguards. There were statistically significant findings in perceived AI usefulness by gender (χ^2^ = 29.2; *p* < 0.001); organizational adoption of AI (χ^2^ = 31.6.2; *p* = 0.047) and where AI is most useful (χ^2^ = 101.1; *p* < 0.001) by qualifications; and support for AI adoption by age (χ^2^ = 18.0; *p* = 0.02). **Conclusions:** U.S. clinicians in our survey viewed GenAI as useful but reported limited training and organizational infrastructure needed for confident use while also expressing concerns regarding data privacy and ethical risk. Education programs and transparent, provider-led implementation strategies may accelerate responsible GenAI assimilation while addressing ethical and workforce concerns. Also, health administrators should use the efficiency gains to improve provider–patient relationships and clinicians’ work–life balance while reducing clinician burnout rates.

## 1. Introduction

Generative Artificial Intelligence (GenAI) has rapidly emerged as a transformative tool in healthcare practice, offering potential advancements in diagnostic accuracy, treatment personalization, operational efficiency, and patient outcomes. GenAI applications now span various areas, including radiology, oncology, cardiology, pathology, and general practice, each utilizing unique AI-driven approaches to improve patient care [1,2,3,4,5,6].

For example, in radiology, AI-powered tools have shown significant utility in image analysis, including evidence of an AI system outperforming radiologists in detecting breast cancer from mammograms, showcasing the potential of AI to enhance diagnostic precision [7]. In oncology, AI algorithms have been developed to predict patient response to specific therapies, optimizing treatment plans [2]. Additionally, in the field of oncology, within the last decade, researchers have found that AI could detect skin cancer with an accuracy comparable to that of dermatologists [8].

In other medical fields, such as cardiology, AI tools have been shown to accurately identify left ventricular dysfunction, which is often challenging for human interpretation, thus aiding in early intervention and patient management [9].

In general practice, AI is increasingly used for tasks such as clinical decision support, patient reports, predictive analytics, and patient monitoring. By analyzing vast datasets from electronic health records (EHRs), GenAI aids clinicians in identifying high-risk patients and guiding treatment decisions, which has become especially relevant in managing chronic diseases like diabetes and hypertension [10].

Despite these advancements, physicians and other healthcare providers often express mixed sentiments about GenAI adoption, citing concerns over reliability, data privacy, and the potential for diminished patient–physician relationships [11]. In 2024 an American Medical Association (AMA) study highlighted that while 36% of physicians felt more excited than concerned about AI; despite a growing majority of physicians recognizing the benefits of AI, with 68% in 2024 reporting at least some advantage in patient care (up from 63% in 2023), our recent global study found AI adoption in healthcare in the United States to be lower than that of Europe [11,12].

Although AI has been widely studied for clinical performance, few studies have investigated U.S. healthcare providers’ perspectives on generative AI adoption, particularly regarding training, workflow integration, and ethical considerations. Understanding and addressing US healthcare providers’ viewpoints can ensure AI tools are designed to support, rather than supplant, the role of providers. This study aimed to describe U.S. healthcare providers’ perceptions of generative AI adoption, including perceived usefulness, training needs, barriers, ethical concerns, and factors influencing willingness to support broader implementation in clinical practice

## 2. Materials and Methods

We conducted a nationwide cross-sectional survey in line with the STROBE checklist for cross-sectional studies (Table S1) using a self-administered questionnaire developed with the Qualtrics electronic data collection tool (https://www.qualtrics.com/) to capture perspectives, beliefs, and opinions on the use of AI in the healthcare sector.

Study Population: The study focused on US healthcare providers such as physicians, nurses, pharmacists, and laboratory scientists. Only healthcare providers currently in practice and working in the United States were eligible and included in the study.

Data Collection Tool: A pretested self-administered questionnaire was used. The pretest was conducted among five (5) providers to validate construct clarity, reduce measurement errors, assess flow and cognitive load, test operational feasibility, evaluate cultural and contextual appropriateness, and improve reliability of the questionnaire. Feedback from participants was used to revise the initial survey tool before its finalization. The questionnaire included sections on AI adoption, deployment, use, benefits, and barriers to AI adoption, as well as basic, anonymized demographic information of the participants. We piloted and reviewed the questionnaire to ensure completeness, accuracy, acceptability, cultural sensitivity, and relevance. Individuals who participated in the pilot were not included in the final study population. The questionnaire and subsequent data analysis complied with relevant protocols and checklists [13].

Sample Size: Sample size estimation followed Cochran’s formula for proportions. Because no robust prevalence figure for GenAI adoption among U.S. clinicians existed when the study was designed, we applied the conservative assumption of *p* = 0.50, a 95% confidence level (Z = 1.96), and a ±5% margin of error, yielding n = 385. Allowing 4% for incomplete surveys produced a target of 402 respondents [14]. A supplementary calculation using the 23% adoption rate [15] indicated a minimum number of 273 participants, confirming that our conservative target of 402 remained adequate.

Sampling and Data Collection Technique: Healthcare providers were identified through a convenience sampling technique of US-based professional organizations, social media platforms, and US member professional networks. The closed, non-randomized survey was sent to 300 healthcare professionals, using personalized emails as well as to their professional network pages. A link to the questionnaire was provided in the email, which required a “one-time only” access to prevent multiple completions of the questionnaire by individual participants.

Data collection occurred over 12 weeks from 1 December 2024 to 28 February 2025. Reminder emails were sent out monthly to prospective participants. The questionnaire was formatted over 20 pages with one to two questions per page and hosted on the Qualtrics website for the duration of the study. Participants were able to check for completeness and could review their answers using a “back button”. If participants were unsure or unwilling to disclose their responses, options including “not sure”, “not applicable”, or “prefer not to say” were available.

Data Analysis: We analyzed data on submitted questionnaires using IBM SPSS version 27 and Microsoft Excel. Analyses use the available-case denominator for each question. Data were uploaded automatically by Qualtrics for analysis. We performed univariate and bivariate analyses. Frequencies, percentages, Chi-square (χ^2^), *p*-value, and degrees of freedom were documented. Comparative analysis was done according to professional role, gender, age, and qualification of the participants. A *p*-value of < 0.05 was deemed to be statistically significant.

Ethical approval was received from the California State University, Dominguez Hills (CSUDH) Institutional Review Board (IRB #: CSUDH IRB-FY2025-98 on 26 November 2024). Participation was voluntary, and no incentives were offered. Written informed consent was obtained from all the subjects prior to study initiation.

## 3. Results

A total of 130 individuals accessed the survey, of whom 109 completed the core and demographic items (completion rate: 83.8%). Among respondents, physicians and nurses represented the largest professional groups. The majority of respondents were aged 50 years or older, held graduate diplomas, and had worked in the health industry for 20 years or more. Participants were primarily Black/African American (38%) and Caucasian (33.8%). The largest proportions of respondents were from California (16.7%) and New York (15.3%). Approximately one quarter were public health specialists (23.6%), and a similar proportion worked in the private sector (23.9%) (Table 1).

### 3.1. Attitude to AI Usefulness in Patient Care and Management

**Figure 1:**
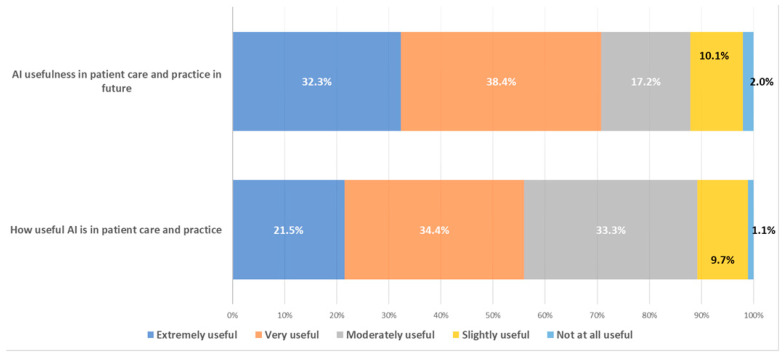
Perceived Current and future usefulness of GenAI in patient care among U.S. clinicians.

Overall, 86.9% of respondents (93/107) believed that AI is useful in patient care and management. Nearly all participants considered AI useful both currently (98.9%) and in the future (98.0%). However, a greater proportion believed AI would be very to extremely useful in the future compared with the present (70.7% vs. 55.9%) (Figure 1). Conversely, more respondents rated AI as moderately useful at present than in the future (33.3% vs. 17.2%). Only a small minority believed AI was not useful in either the present (1.1%) or the future (2.0%).

Male participants were significantly more likely to believe that AI has a role in patient care and management (χ^2^ = 29.2, *p* < 0.001) (Table 2). No statistically significant differences were observed by professional role, age, or qualifications

### 3.2. Training and Adoption of AI

Less than half of respondents have had formal exposures or training on AI (42.4%)), and this was a basic orientation to AI (83.3%), AI use in patient care (31%, 13/42), or technical aspects of AI (33.3%, 14/42). While 23.2% of respondents’ organizations have officially adopted AI, 38.4% (38/99) have trained staff on AI. Most adoption processes are led by top-level/executive leadership (32.8%, 20/61), although 24.6% (15/61) of participants were unaware of who was leading the adoption process (Table 3). Organizations of participants holding doctorate degrees were statistically more likely to adopt AI when compared with participants with other qualifications (χ^2^ = 31.6; *p* = 0.047) (Table 2). However, there were no statistically significant differences by professional roles, gender or age of participants.

### 3.3. AI Use in Patient Care and Management

AI was mostly used in report writing (43.1%, 28/65), research (27.7%, 18/65), patient care (26.2%, 17/65), and diagnosis (24.6%, 16/65). AI was also used in leadership and management (21.5%, 14/65). However, in patient care, AI was mostly useful in time management and documentation activities (34.2%, 25/73), and to improve patient registration processes and research (20.5%, 15/73) (Table 4). Participants holding a doctorate degree were statistically more likely to identify patient diagnosis and report writing as areas where AI was most useful when compared to participants with other qualifications (χ^2^ = 101.1; *p* < 0.001).

### 3.4. Challenges and Barriers to AI Adoption and Use

While poor knowledge of AI (24.7%, 19/77) and fear of job loss (16.9%, 13/77) were the leading barriers to AI adoption and use, 72.2% (57/79) of providers were willing to support AI adoption in clinical care. Other identified barriers included cost of acquisition of AI, staff skills and capacities, staff resistance to change, leadership and management issues, inadequate technology and equipment, and limited interest and or negative attitude of staff. Key patient care practice challenges included lack of human oversight (58.2%, 46/79), bias in AI algorithms and overdependence on AI (54.4%, 43/79). Others were unintended consequences and ethical/legal challenges (48.1% [38/79] and 41.8% [33/79], respectively) (Table 5). Participants who were less than 50 years of age were statistically more likely to support AI adoption and embedding in organizations (χ^2^ = 18.0; *p* = 0.02). However, there were no statistically significant differences by professional roles, gender or qualifications of participants.

However, strategies identified by participants to mitigate challenges of AI in healthcare include (but are not limited to) the absence of human oversight (67.1%, 53/79), poor staff training 60.8%, 48/79, and lack of provider involvement in design and development of AI tools and resources (57.0%, 45/79) (Table 5).

### 3.5. Core Benefits and Ethical Issues Relating to AI in Healthcare

In practice, participants believed that the most important benefit of AI was in patients’ documentation and clerking (57.0%, 45/79), as it minimizes errors and mistakes (49.4%, 39/79). Privacy and surveillance issues (63.3%, 50/79) and security risks (55.7%, 44/79) were identified as the most important ethical issues associated with the use of AI in healthcare (Table 6).

### 3.6. Impact of AI Adoption Integration on Clinicians’ Workload

While 17.1% (13/76) of respondents were willing to support an increase in providers’ patient load due to AI efficiency gains, the rest were either against it (38.2%, 29/76) or undecided (maybe, 44.7%, 34/76).

## 4. Discussion

Our survey of healthcare professionals practicing in the United States found that healthcare professionals perceive AI as useful in patient care and management, despite fewer than half having received formal training in AI. Several barriers to AI adoption were identified, including limited knowledge of AI and concerns about job displacement. Nevertheless, most respondents indicated a willingness to support AI integration in clinical care, suggesting that targeted education and capacity-building initiatives may help address existing concerns. Participants also emphasized the importance of human oversight, improved staff training, and greater provider involvement in the design and development of AI tools to ensure safe and effective implementation. Ethical concerns related to privacy, surveillance, and data security were noted. 

Recent studies have shown a significant uptake in the use of GenAI in clinical practice among physicians and other providers. The AMA Augmented Intelligence Research involving 1183 physicians revealed that a growing majority of physicians are beginning to recognize the benefits of AI, especially the advantages in in patient care [11]. On the heels of this finding, our study reveals that between 87% (current) and 93% (future) of healthcare providers who participated in the study believe that GenAI is at least moderately useful in patient care in the present and in the future. This is a massive acceptance rate, showing that AI may have come to stay in healthcare and patient management. The substantial growth in physician use of AI in practice, with the usage of AI nearly doubling from 2023 to 2024 and a dramatic drop in non-users in just one year in the AMA study, supports this assertion. Furthermore, the very high rate of AI acceptance in our study in less than a year after the AMA study shows a continued improvement in providers’ acceptance and use of AI in clinical care. However, providers still have their doubts, issues, and fears regarding AI that could be minimized by proper training, formal exposure, and continued top management support and use of AI. These fears are similar to recent findings in another global study including US providers [12].

Providers have significant faith in GenAI, understand where AI is most useful and some current barriers/concerns. However, less than half have been formally trained or exposed to AI, and less than a quarter of participants’ organizations have adopted AI. To advance GenAI in healthcare, training for providers is imperative. Formal training of healthcare leaders will also help them to become advocates for AI adoption and embedding in healthcare systems as it will expose them to the benefits of AI in healthcare systems. With proper guidance and a better understanding of human-in-the-loop AI development and deployment strategies, providers will better accept human-supervised GenAI as safe, reproducible, reliable and significantly accurate, and understand that AI is not positioned to take over their jobs. This will motivate more providers to venture into GenAI-supported patient care and health management.

Like the AMA study findings where 68% of physicians believed that AI has some or definite advantages in patient care, our study revealed that GenAI has significant advantages in patient care, especially in patient documentation and report writing. Also, our findings revealed that significantly more providers are currently using AI for patient documentation processes and report writing, discharge summaries and care plans, and medical research and standard of care summaries, similar to AMA findings [11]. The current increase in clinicians’ use may be attributed to accelerated AI adoption in healthcare during the COVID-19 pandemic, which may have influenced clinicians’ perception by highlighting AI’s practical utility in crisis response [16,17,18]. Also, AI’s perceived usefulness and clinical value with evidence of performance, improved transparency and explainability, perceived ease of use, regulation and governance systems, improved data quality and security, organizational and social influence, and specialty and task fits may all have contributed directly or otherwise to improved adoption and use by clinicians [19,20,21,22,23,24,25,26,27]. Also, the current sociocultural and economic contexts, including large-scale investments in AI technology and increasing public awareness of AI, could partly account for the external factors shaping healthcare workers’ attitudes to AI in health [28,29,30,31,32].

However, the integration of GenAI in healthcare comes with both opportunities and challenges, as AI adoption in healthcare has significantly improved diagnostic accuracy, streamlined workflow processes, and enhanced patient care, including personalized treatment [33]. Despite major advances in AI research for healthcare, the deployment and adoption of AI technologies remain limited in clinical practice, thus the need for more formal training, on the job mentoring, and clarity on their use [34,35]. The increased use of AI in health and the expanding science around AI in clinical medicine have not eliminated the concerns people have, requiring that developers and users prioritize patient-relevant outcomes to fully understand AI’s true effects and limitations in healthcare [36]. Also, providers should play a significant role in AI design, development and deployment through in and out innovation approaches [12,37,38]

Our study outcomes support previously identified findings that challenges such as ethical and legal concerns, patient privacy, and data security remain prominent obstacles hindering providers’ adoption of AI [12,37,38,39] as the continued use of GenAI tools and resources increases the risk of unauthorized data breaches. Beyond data-breach risks, 63% of clinicians expressed unease about GenAI-enabled surveillance and the continuous algorithmic monitoring of both patients and providers, which they felt could erode autonomy and trust, unless strict transparency, opt-in consent, and usage boundary safeguards are put into place. This calls for better data security and improved use of firewalls and relevant tools to protect against patient data breaches. To minimize these concerns, providers and healthcare organizations must promote a culture of transparency, accountability, and openness as to the capacity, potential and use of GenAI, and ensure vigilance over patient data security. While waiting for relevant policies and guidelines, there is an urgent need for developers and users alike to address these ethical, technical, and security challenges that GenAI brings. Ensuring that due attention is paid to the ETHICS (environmental concerns, transparency and explainability, hallucinations, inclusiveness and inconsistencies, cost and clinical workflow integration, and safety and security of data) of AI, and that appropriate policies govern all adoption initiatives, will greatly minimize these concerns [39].

Additionally, approximately 39.2% of participants identified algorithmic transparency (the “Black Box” problem) as a key challenge in adopting generative AI. Incorporating explainable AI (XAI) approaches, which provide interpretable outputs and reasoning behind model predictions, could help increase provider trust and facilitate clinical integration. Future work should explore how XAI tools can be implemented in healthcare workflows to address transparency concerns.

Therefore, to effectively navigate the path forward to realize the potential of GenAI in healthcare and health, there is an urgent need to ensure appropriate skill generation; model testing, implementation, and monitoring; resources and infrastructure; and standardized oversight and guidelines [40]. Additional large-scale multiple-site studies that will explore in depth the findings of this study are needed using a deliberate proactive strategy [41]. Additionally, the fact that 16.9% are afraid of losing their jobs, but only 17.1% would be willing to accept an increased patient load because of the AI productivity gain calls for a qualitative study that will look into the issues of providers’ burnout and the willingness of healthcare managers to reinvest the saved time in advancing better patient–provider relationships.

## 5. Limitations

We could not achieve our calculated sample size due to providers’ inability to allocate the time needed to complete the questionnaires. With 130 completed responses, the survey achieved a 95% confidence interval of ±8.6% around a 50% proportion, wider than the ±5% originally planned. The reduced sample size likely decreased the statistical power of the study and increased the risk of Type II error, particularly for analyses using the Chi-square test to examine associations between categorical variables. The convenience sampling approach resulted in an overrepresentation of providers from California and New York, as well as certain racial demographic groups. Because attitudes toward emerging technologies may vary across geographic and demographic contexts, these concentrations could have influenced the relatively high acceptance rates of AI observed in this sample. Therefore, the generalizability of our findings is not guaranteed for other demographics and regions of the country. Also, we do not have complete data on some participants who started the process but did not fully complete the questionnaire. Their experiences and views may be different from those who completed the questionnaire. The study is also subject to the challenges of online surveys, including selection bias as only providers within the authors’ personal and professional networks were invited to participate in the survey. Because recruitment relied on authors’ networks and listservs, the sample is not probabilistic, and over-represents providers from California and New York, as well as Black/African Americans. Findings should therefore be interpreted as hypothesis-generating, rather than nationally representative. Future research should examine AI adoption using larger and more nationally representative samples of U.S. healthcare providers, as well as longitudinal designs to better understand how perceptions and use of generative AI evolve over time. Additional work is also needed to identify effective strategies for addressing data privacy concerns, strengthening governance frameworks, and improving training and workforce preparedness for AI integration in healthcare.

## 6. Conclusions

Healthcare providers have a high GenAI acceptance rate, and there is poor formal training for providers and other users. There is an urgent need to develop an AI-In-Service training curriculum and expose US providers formally to GenAI. This shortfall in formal instruction is reflected in our data, as ‘limited knowledge of AI’ was the single most cited barrier, indicating that structured, hands-on training could directly mitigate clinicians’ knowledge gaps and increase adoption readiness. Also, healthcare managers should be more transparent and communicative on their GenAI adoption initiatives and share the process, experiences, challenges, and successes with their teams.

## Figures and Tables

**Table 1:** Demographic information of respondents.

Description	Frequency	Percentage
***Role in Healthcare*** (***n = 109***)		
Physician	42	38.50%
Nurse	18	16.50%
Allied Healthcare Professional	14	12.80%
Hospital Administrator	3	2.80%
Other Providers	32	29.40%
***Gender at Birth*** (***n = 72***)		
Prefer not to say	2	2.80%
Female	39	54.20%
Male	31	43.10%
***Age of Respondents*** (***n = 71***)		
60 years and above	19	26.80%
50–59 years	27	38.00%
40–49 years	9	12.70%
30–39 years	14	19.70%
20–29 years	2	2.80%
***Highest Education*** (***n = 71***)		
Doctorate	39	54.90%
Masters	17	23.90%
Bachelors	11	15.50%
High School Diploma/GED	2	2.80%
Others	2	2.80%
***Length in Health Industry*** (***n = 71***)		
25 or more years	33	46.50%
20–24 years	12	16.90%
15–19 years	4	5.60%
10–14 years	9	12.70%
5–9 years	10	14.10%
Less than 5 years	3	4.20%
***Current Work location*** (***n = 71***)		
Private	17	23.90%
Nonprofit/Public Charity	12	16.90%
College/University	14	19.70%
County/Local	7	9.90%
Federal or State	10	14.10%
Others	11	15.50%
***Current work Section*** (***n = 72***)		
Public Health and Preventive Medicine	17	23.60%
Internal Medicine	10	13.90%
Family Medicine	8	11.10%
Pediatrics	7	9.70%
Pathology	3	4.20%
Psychiatry	3	4.20%
Geriatrics	2	2.80%
Ophthalmology	2	2.80%
Obstetrics and Gynecology	2	2.80%
Surgery	1	1.40%
***Race or Ethnicity*** (***n = 71***)		
Black/African American	27	38.00%
White/Caucasian	24	33.80%
Hispanic/Latino/Latinx	7	9.90%
Native American/Alaska Native	1	1.40%
Pacific Island/Hawaii	0	0.00%
East Asian	5	7.00%
South Asian	4	5.60%
Arab/Middle Eastern	2	2.80%
Mixed	0	0.00%
I prefer not to say	7	9.90%

**Table 2:** Chi-Square (χ^2^) analysis of participants’ responses to AI use, adoption, and staff training.

Description	Professional Role		Gender		Age		Qualification	
	Chi-Square (χ^2^)	*p*-Value (df)	Chi-Square (χ^2^)	*p*-Value (df)	Chi-Square (χ^2^)	*p*-Value (df)	Chi-Square (χ^2^)	*p*-Value (df)
Artificial Intelligence (AI) has a role or usefulness in patient care and management	9.3	0.32 (8)	**29.2**	**<0.001** (**4**)	7.7	0.46 (8)	3.5	0.97 (10)
AI usefulness in the future in patient care and practice	14.1	0.59 (16)	7.1	0.31 (6)	12.7	0.39 (12)	11.6	0.71 (15)
Have had formal exposure or training in AI	1.2	0.99 (8)	0.3	0.99 (4)	7.8	0.45 (8)	8.7	0.57 (10)
The organization has adopted/begun the process of adopting AI	18.8	0.28 (16)	6.0	0.64 (8)	23.3	0.06 (16)	**31.6**	**0.047** (**20**)
The organization has trained someone in AI use	7.2	0.51 (8)	0.9	0.92 (4)	14.8	0.06 (8)	6.3	0.79 (10)
Where AI is most useful in the healthcare industry	32.5	0.90 (44)	22.7	0.42 (22)	28.5	0.97 (44)	**101.1**	**<0.001** (**55**)
Where AI is least useful in the healthcare industry	42.5	0.54 (44)	12.9	0.94 (22)	41.4	0.58 (44)	56.3	0.43 (55)
The most important barrier to AI adoption and implementation in patient care	36.4	0.45 (36)	8.6	0.48 (9)	37.2	0.41 (36)	29.6	0.96 (45)
Will support AI adoption and embedding in the organization	8.2	0.42 (8)	6.2	0.18 (4)	**18.0**	**0.02** (**8**)	11.4	0.33 (10)

Note: Bild signifies statistical differences.

**Table 3:** AI adoption and embedding in the organization.

Description	Freq	Percentage
***AI is useful in-patient care and management*** (***n = 107***)		
Yes	93	86.90%
Neither true nor false	9	8.40%
No	5	4.70%
***How useful AI is in patient care and practice*** (***n = 93***)		
Extremely useful	20	21.50%
Very useful	32	34.40%
Moderately useful	31	33.30%
Slightly useful	9	9.70%
Not at all useful	1	1.10%
***AI usefulness in patient care and practice in the future*** (***n = 99***)		
Extremely useful	32	32.30%
Very useful	38	38.40%
Moderately useful	17	17.20%
Slightly useful	10	10.10%
Not at all useful	2	2.00%
***Formal exposure or training in AI*** (***n = 99***)		
Not Sure	5	5.10%
No	52	52.50%
Yes	42	42.40%
***Training individuals were exposed to*** (***n = 42***)		
Basic orientation to AI	35	83.30%
Training on AI use in patient care (diagnosis, treatment, etc.)	13	31.00%
Training in AI use in management and leadership	11	26.20%
Training in technical aspects of AI	14	33.30%
Other forms of AI training	11	26.20%
***Organization has adopted/begun the process of AI adoption*** (***n = 99***)		
I do not know	11	11.10%
No, we have not started adopting AI	37	37.40%
Yes, we are beginning to think about adopting AI	24	24.20%
Yes, we will adopt AI	4	4.00%
Yes, we have adopted AI	23	23.20%
***Leaders of AI adoption in Organizations*** (***n = 61***)		
Others (Please specify)	4	6.60%
I do not know	15	24.60%
Outsourced	5	8.20%
IT Staff	8	13.10%
Administration Staff	5	8.20%
Middle Level/Management Staff	4	6.60%
Top-level/Executive Leadership	20	32.80%
***Organizational training on AI use*** (***n = 99***)		
I do not know/I am not sure	38	38.40%
No	36	38.40%
Yes	25	38.40%

**Table 4:** Acceptance and use of AI by healthcare providers.

Description	Freq	Percentage
***Where AI is commonly used*** (***n = 65***)		
Report writing	28	43.10%
Research	18	27.70%
Patient care (e.g., treatment, continuity of care, referral, etc.)	17	26.20%
Diagnosis (e.g., radiology, pathology, endoscopy, etc.)	16	24.60%
Leadership and management	14	21.50%
Strategic management	12	18.50%
Staff and personnel management	9	13.80%
Resource management	8	12.30%
Precision medicine (e.g., gene therapy, cancer management, etc.)	6	9.20%
I do not want to specify	6	9.20%
Others	14	21.50%
***Aspects of patient care where AI is most useful*** (***n = 73***)		
Time management	25	34.20%
Documentation activities	25	34.20%
Improved patient registration processes	15	20.50%
Research	15	20.50%
Diagnosis	13	17.80%
Team management	11	15.10%
Patient management and care	10	13.70%
Errors and mistakes	10	13.70%
Continuity of care and follow up processes	10	13.70%
Patient clerking and history taking	9	12.30%
Provider’s personal job satisfaction	9	12.30%
Laboratory processes	8	11.00%
Prescription practices	7	9.60%
Provider burnout of providers	5	6.80%
Work–life balance	5	6.80%
Provider health and wellbeing	4	5.50%
Patient satisfaction	3	4.10%
Others	19	26.00%
***Healthcare activity where AI is very useful*** (***n = 77***)		
Report writing	19	24.70%
Diagnosis (e.g., radiology, pathology, endoscopy, etc.)	18	23.40%
Strategy development	7	9.10%
Patient care (e.g., treatment, continuity of care, referral, etc.)	7	9.10%
None of the above	6	7.80%
Leadership and management	5	6.50%
Precision medicine (e.g., cancer management)	4	5.20%
Resource management	3	3.90%
I do not want to specify	2	2.60%
Financial management	2	2.60%
Staff management	1	1.30%
Others	3	3.90%

**Table 5:** Barriers to AI use and mitigation strategies.

Descriptions	Freq	Percentage
***Most important barrier to AI adoption and implementation in patient care*** (***n = 77***)		
Knowledge of AI	19	24.70%
Fear of job loss	13	16.90%
Cost of acquisition	8	10.40%
Staff skills and capacities	7	9.10%
Organization-wide adoption of AI	6	7.80%
Staff resistance to change	5	6.50%
Leadership and management	4	5.20%
Technology and equipment	4	5.20%
Interest and attitude of staff	1	1.30%
Others	8	10.40%
***Willingness to support AI adoption and embedding*** (***N = 79***)		
Not sure	17	22.50%
No	5	6.30%
Yes	57	72.20%
***Patient care practice challenges*** (***n = 79***)		
Lack of human oversight	46	58.20%
Bias in AI algorithms	43	54.40%
Overdependence on AI	43	54.40%
Unintended consequences	38	48.10%
Ethical and legal challenges	37	46.80%
Data privacy and security concerns	33	41.80%
Algorithmic opacity (Black Box problems)	31	39.20%
Job displacement	26	32.90%
Reduced patient–provider interaction	25	31.60%
More workload	19	24.10%
High cost and accessibility issues	17	21.50%
***Strategies to mitigate the challenges of AI in healthcare*** (***N = 79***)		
Human oversight	53	67.10%
Staff training	48	60.80%
Provider involvement in design and development	45	57.00%
Data protection	39	49.40%
Enhanced transparency	34	43.00%
Improved accessibility	25	31.60%
Early adoption and integration	23	29.10%

**Table 6:** Core benefits and ethical issues relating to AI use in healthcare.

Description	Freq	Percentage
***Core benefits of AI in clinical practice*** (***n = 79***)		
Facilitates patients’ documentation and clerking	45	57.00%
Minimizes errors and mistakes	39	49.40%
Opens up time for better provider–patient communication	34	43.00%
Shortens turnaround time for requests	34	43.00%
Improves provider–patient relationship	13	16.50%
Encourages provider–patient relationship	12	15.20%
Others	9	11.40%
***Ethical issues associated with AI use in healthcare*** (***n = 79***)		
Privacy and surveillance	50	63.30%
Security risks	44	55.70%
Misinformation and deepfakes	40	50.60%
Lack of regulations and polices	40	50.60%
Bias and fairness	37	46.80%
Autonomy and decision making	32	40.50%
Ethical use in education and patient care	30	38.00%
Ownership and intellectual property	30	38.00%
Job displacement and economic impact	27	34.20%
Transparency and accountability	22	27.80%

## Data Availability

Data supporting the reported results can be obtained upon request from the first author.

## Supplementary Materials

The following supporting information can be downloaded at: https://www.mdpi.com/article/10.3390/healthcare14060775/s1, Table S1: Strobe Checklist; File S1: Survey questions.

## Abbreviations

The following abbreviations are used in this manuscript:

AI	Artificial Intelligence
AMA	American Medical Association
EHR	Electronic Health Records
GenAI	Generative Artificial Intelligence
SPSS	Statistical Package for Social Sciences
