# Supplementary material for: Healthcare Providers’ Perspectives on Generative Artificial Intelligence (GenAI) Adoption, Adaptation, Assimilation, and Use in the United States
Source: Healthcare (Basel). 2026 Mar 19;14(6):775. doi: 10.3390/healthcare14060775 (PMC13027095; doi:10.3390/healthcare14060775)
Supplement: Supplementary file 1 [file healthcare-14-00775-s001.zip › healthcare-4175651-Supplementary File S1_Questionnaire with Consent Form.pdf]

Providers' Perspectives on AI Adoption, Use and Next Steps

Start of Block: Default Question Block

California State University, Dominguez Hills Study Information Sheet Introduction: You are being asked to participate in a research study conducted by Obinna Oleribe (DrPH, MBA, MBBS) from the School of Public Health and Health Sciences, College of Health, Human Services and Nursing at California State University, Dominguez Hills. As a healthcare provider, you are invited to participate in this study as your participation will help generate a broad and better understanding of the concepts.
Purpose and Description of the Study: The purpose of this study is to document the perspectives, knowledge, attitudes, and practices of physicians to artificial intelligence (AI). If you decide to participate in this study, you will complete a self-administered questionnaire (survey). The survey has questions that explore your knowledge, understanding, and use of AI. We will also collect some demographic information, but no personally identifiable data will be collected. The survey should take approximately 10 minutes to complete.
Risk(s) and Discomfort(s): There are no risk(s) associated with this study. Participation in this study is voluntary and if you choose to participate in this study, you may withdraw at any time without any consequences. You may also refuse to answer any questions you don't want to answer and still remain in the study.
Benefits: By participating in this study, you will contribute to science, advance AI evolution, and provide vital information for evidence-based decisions.
Confidentiality: The study is confidential, so any information provided cannot be traced back to you. Please do not include your name or other identifying information in your survey responses that can identify you. Data will be analyzed in aggregate and stored in a secure location outside the reach of non-investigators.
Contact information: If you have any questions or concerns about the research, please feel free to contact Obinna Oleribe at *ooleribe@csudh.edu*. If you have questions regarding your rights as a research participant, contact the California State University, Dominguez Hills IRB Office at 310-243-3756 or *irb@csudh.edu.*
This study information sheet is for you to keep. Please save or print a copy of this page for your records or take a screenshot of it. Do you agree to participate

○ Yes (1)

○ No (2)

Skip To: End of Survey If California State University, Dominguez Hills Study Information Sheet Introduction: You are being... = No

Q0 I am a....

◯ Physician (1)

◯ Nurse (2)

◯ Other providers (please specify) (4)
____

◯ None of the above (5)

Skip To: End of Survey If I am a.... = None of the above

Q1 Do you think Artificial Intelligence (AI) has any role or usefulness in patient care and management?

◯ Yes (1)

◯ No (2)

◯ Neither true nor false (3)

Skip To: Q2 If Do you think Artificial Intelligence (AI) has any role or usefulness in patient care and management? = No

Q1a If yes, how useful do you think AI is in patient care and practice?

◯ Not at all useful (1)

◯ Slightly useful (2)

◯ Moderately useful (3)

◯ Very useful (4)

◯ Extremely useful (5)

Q2 In the future, how useful do you think AI will be in patient care and practice?

- Not at all useful (1)
- Slightly useful (2)
- Moderately useful (3)
- Very useful (4)
- Extremely useful (5)

Q3 Have you had any formal exposure or training in AI?

- Yes (1)
- No (2)
- Not Sure (3)

Skip To: Q4 If Have you had any formal exposure or training in AI? = No

Q3a If yes, what kind of training or exposure did you have (Tick all that apply)?

- Basic Orientation to AI (1)
- Training on AI use in patient care (diagnosis, treatment, lab services, etc.) (2)
- Training in AI use in management and leadership (3)
- Training in technical aspects of AI (4)
- Other forms of AI training (5)

Q4 Has your organization adopted/begun the process of AI adoption, adaptation, and use?

- Yes, we have adopted AI (1)
- Yes, we will adopt AI (2)
- Yes, we are beginning to think about adopting AI (3)
- No, we have not started adopting AI (4)
- I do not know (5)

Display This Question:

If Has your organization adopted/begun the process of AI adoption, adaptation, and use? != No, we have not started adopting AI

Q4a If your organization has started adopting AI, who is leading the process of AI adoption?

- Top-level/Executive Leadership (1)
- Middle Level/Management Staff (2)
- Operational Staff (3)
- Technical Staff (4)
- Outsourced (5)
- I do not know (6)

Q5 Has your organization trained anyone on AI use?

- Yes (1)
- No (2)
- I do not know/I am not sure (3)

Display This Question:

If Has your organization trained anyone on AI use? = Yes

Q5a If staff has been trained, who benefited from the training (Tick all that apply)?

- ☐ Executive Leadership/Top-Level (1)
- ☐ Management Staff/Mid-level (2)
- ☐ Operational Staff (3)
- ☐ IT staff (4)
- ☐ Others (5)
- ☐ I do not want to answer (6)

Q6 Where is AI commonly used in your organization?

- Patient care (e.g. Treatment, Continuity of care, Referral, etc.) (1)
- Diagnosis (e.g. Radiology, Pathology, Endoscopy, etc.) (2)
- Precision Medicine (e.g. Gene Therapy, Cancer management, etc.) (3)
- Leadership and management (4)
- Strategic management (5)
- Resource management (6)
- Staff and personnel management (7)
- Report Writing (8)
- Research (13)
- None of the above (9)
- I do not want to specify (10)
- Others (Please Specify) (11) ____

Q7 Which aspects of patient care has AI POSITIVELY impacted in your organization? (Tick all that apply)

- ☐ Improved patient registration processes (1)
- ☐ Patient clerking and history taking (9)
- ☐ Diagnosis (2)
- ☐ Patient management and care (3)
- ☐ Time management (8)
- ☐ Errors and mistakes (4)
- ☐ Prescription practices (6)
- ☐ Continuity of care and follow up processes (5)
- ☐ Laboratory processes (7)
- ☐ Provider burnout of providers (10)
- ☐ Documentation activities (11)
- ☐ Patient satisfaction (15)
- ☐ Team management (16)
- ☐ Provider health and wellbeing (17)
- ☐ Work Life Balance (18)
- ☐ Research (20)
- ☐ Provider's personal job satisfation (19)

☐ Others (Please Specify) (14) ________________

Q8 Which aspects of patient care has AI NEGATIVELY impacted in your organization? (Tick all that apply)

- ☐ Improved patient registration processes (1)
- ☐ Patient clerking and history taking (9)
- ☐ Diagnosis (2)
- ☐ Patient management and care (3)
- ☐ Time management (8)
- ☐ Errors and mistakes (4)
- ☐ Prescription practices (6)
- ☐ Continuity of care and follow up processes (5)
- ☐ Laboratory processes (7)
- ☐ Provider burnout of providers (10)
- ☐ Documentation activities (11)
- ☐ Patient satisfaction (15)
- ☐ Team management (16)
- ☐ Provider health and wellbeing (17)
- ☐ Work Life Balance (18)
- ☐ Provider's personal job satisfation (19)
- ☐ Others (Please Specify) (14)

Q9 Where is AI MOST useful in the healthcare industry?

- Patient care (e.g. Treatment, Continuity of Care, Referral, etc.) (1)
- Diagnosis (e.g. Radiology, Pathology, Endoscopy, etc.) (2)
- Precision medicine (e.g. cancer management) (9)
- Leadership and management (3)
- Financial management (4)
- Staff management (5)
- Resource management (6)
- Report writing (7)
- Strategy development (8)
- None of the above (10)
- I do not want to specify (11)
- Others (Please Specify) (12) ____

Q10 Where is AI LEAST useful in the healthcare industry?

- Patient care (e.g. Treatment, Continuity of Care, Referral, etc.) (1)
- Diagnosis (e.g. Radiology, Pathology, Endoscopy, etc.) (2)
- Precision medicine (e.g. cancer management) (9)
- Leadership and management (3)
- Financial management (4)
- Staff management (5)
- Resource management (6)
- Report writing (7)
- Strategy development (8)
- None of the above (10)
- I do not want to specify (11)
- Others (Please Specify) (13) ____

Q11 What is the MOST important barrier to AI adoption and implementation in patient care?

- Fear of job loss (9)
- Cost of acquisition (5)
- Knowledge of AI (3)
- Staff resistance to change (2)
- Interest and attitude of staff (1)
- Staff skills and capacities (4)
- Technology and equipment (6)
- Leadership and management (7)
- Organization wide adoption of AI (8)
- Others (Please Specify) (10) ____

Q13 Will you support AI adoption and embedding in your organization?

- Yes (1)
- No (2)
- Not sure (4)

Display This Question:

If Will you support AI adoption and embedding in your organization? != Yes

Q13a If your answer above is "No" or "Not sure", why?

____

Q14 What are the core benefits of AI in clinical practice? (Tick all that apply)

- ☐ Encourages provider patient relationship (6)
- ☐ Opens up time for better provider patient communication (7)
- ☐ Shortens turnround time for requests (8)
- ☐ Minimizes errors and mistakes (4)
- ☐ Improves provider patient relationship (5)
- ☐ Facilitates patients' documentation and clerking (9)
- ☐ Others (Please Specify) (10) ____

Q42 Some managers are already increasing the patient loads of providers due to better time management, should managers increase the patient load of providers following the improved efficiency?

- ○ No (18)
- ○ Maybe (19)
- ○ Yes (20)

Q33 What ethical issues are you worried about in relation to AI use in healthcare? (Tick all that apply)

- ☐ Privacy and Surveillance (1)
- ☐ Transparency and Accountability (2)
- ☐ Bias and Fairness (3)
- ☐ Autonomy and Decision Making (4)
- ☐ Job Displacement and Economic Impact (5)
- ☐ Ethical use in Education and Patient care (6)
- ☐ Misinformation and Deepfakes (7)
- ☐ Lack of regulations and Polices (8)
- ☐ Ownership and Intellectual Property (9)
- ☐ Security Risks (10)
- ☐ Others (Please Specify) (11) ____

Q34 What patient care practice challenges are you worried about?

- ☐ More workload (1)
- ☐ Bias in AI Algorithms (2)
- ☐ Lack of Human Oversight (3)
- ☐ Data Privacy and Security Concerns (4)
- ☐ Reduced Patient-Provider Interaction (5)
- ☐ Algorithmic Opacity (Black Box problems) (6)
- ☐ Overdependence on AI (7)
- ☐ High Cost and Accessibility Issues (8)
- ☐ Job Displacement (9)
- ☐ Ethical and Legal Challenges (10)
- ☐ Unintended Consequences (11)
- ☐ Others (Please specify) (12) ____

Q35 How best can we mitigate the challenges of AI in healthcare?

- ☐ Human Oversight (1)
- ☐ Staff Training (2)
- ☐ Enhanced Transparency (3)
- ☐ Data protection (4)
- ☐ Provider involvement in design and development (5)
- ☐ Improved accessibility (6)
- ☐ Early adoption and integration (7)
- ☐ Others (Please specify) (8) ____

End of Block: Default Question Block

Start of Block: Demographic Information: Now let us get to know you better.

Q15 What was your gender at birth?

- ○ Male (1)
- ○ Female (2)
- ○ Non-binary / third gender (3)
- ○ Others (4)
- ○ Prefer not to say (5)

Q16 How old are you in completed years?

- Less than 20 years (1)
- 20 - 29 years (2)
- 30 - 39 years (3)
- 40 - 49 years (4)
- 50 - 59 years (5)
- 60 years and above (6)

Q17 What is your highest educational qualification?

- High School Diploma/GED (1)
- Bachelors (2)
- Masters (3)
- Doctorate (4)
- Others (5)
- I do not want to specify (6)

Q18 How long have you been working in the health industry?

- Less than 5 years (1)
- 5 - 9 years (2)
- 10 - 14 years (3)
- 15 - 19 years (4)
- 20 - 24 years (5)
- 25 or more years (6)

Q19 Where are you currently working?

- Federal or State (1)
- County/Local (2)
- College/University (3)
- Non Profit/Public Charity (4)
- Private (5)
- Others (Please Specify) (6) ____

Q20 In which section are currently working?

- Internal Medicine (13)
- Pediatrics (14)
- Obstetrics and Gynecology (OB-GYN) (15)
- Surgery (16)
- Psychiatry (17)
- Neurology (18)
- Dermatology (19)
- Emergency Medicine (20)
- Anesthesiology (21)
- Ophthalmology (22)
- Otolaryngology (ENT) (23)
- Orthopedics (24)
- Radiology (25)
- Pathology (26)
- Public Health and Preventive Medicine (28)
- Geriatrics (29)
- Family Medicine (30)
- Others (Please Specify) (27) ____

Q21 What is your race or ethnicity?

- ○ Black/African American (1)
- ○ White/Caucasian (2)
- ○ Hispanic/Latino/Latinx (3)
- ○ Native American/Alaska Native (4)
- ○ Pacific Island/Hawaii (6)
- ○ East Asian (7)
- ○ South Asian (10)
- ○ Arab/Middle Eastern (11)
- ○ Mixed (5)
- ○ I prefer not to say (9)
- ○ Others (12)

Q22 In which state are you currently located?

▼ Alabama (8) ... Wyoming (67)

Q23 What additional function/task(s) would you like AI to have?

____

End of Block: Demographic Information: Now let us get to know you better.
